# Citalopram, an antipsychotic agent, induces G1/G0 phase cell cycle arrest and promotes apoptosis in human laryngeal carcinoma HEP-2 cells

**DOI:** 10.1007/s12032-024-02338-0

**Published:** 2024-04-04

**Authors:** Mohammed Salama, Abeer Ali, Fawziya A. R. Ibrahim, Seham Elabd

**Affiliations:** 1 Department of Histochemistry and Cell Biology, Medical Research Institute, Alexandria University, 165 El-Horeya Avenue, Hadara, Alexandria Egypt; 2 Department of Chemical Pathology, Medical Research Institute, Alexandria University, 165 Horreya Avenue, Hadara, Alexandria Egypt; 3 Department of Applied Medical Chemistry, Medical Research Institute, Alexandria University, 165 Horreya Avenue, Hadara, Alexandria Egypt; 4 Department of Physiology, Medical Research Institute, Alexandria University, 165 Horreya Avenue, Hadara, Alexandria Egypt

**Keywords:** Citalopram, Laryngeal carcinoma, HEP-2 cells, Apoptosis, Cell cycle arrest

## Abstract

Human laryngeal squamous carcinoma (LSCC) is a common malignant tumor in the head and neck. Despite the recently developed therapies for the treatment of LSCC, patients’ overall survival rate still did not enhance remarkably; this highlights the need to formulate alternative strategies to develop novel treatments. The antitumor effects of antidepressant drugs such as citalopram have been reported on several cancer cells; however, they have yet to be investigated against LSCC. The current study was directed to explore the possible antitumor effects of citalopram on human laryngeal carcinoma cell lines (HEP-2). HEP-2 cells were cultured and treated with different doses of citalopram (50–400 µM) for 24, 48, and 72 h. The effects of citalopram on the viability of cancer cells were determined by the MTT assay. In addition, apoptosis and cell cycle analysis were performed by flow cytometry. Moreover, evaluation of the expression of proapoptotic and apoptotic proteins, such as cytochrome c, cleaved caspases 3 and 9, Bcl-2, and BAX, was performed by western blotting analysis. Our results revealed that citalopram significantly suppressed the proliferation of HEP-2 cells through the upregulation of p21 expression, resulting in the subsequent arrest of the cell cycle at the G0/G1 phase. Furthermore, citalopram treatment-induced HEP-2 cell apoptosis; this was indicated by the significant increase of cytochrome c, cleaved caspases 3 and 9, and BAX protein expression. On the contrary, Bcl-2 protein expression was significantly downregulated following treatment with citalopram. The ultrastructure studies were in accordance with the protein expression findings and showed clear signs of apoptosis with ring chromatin condensation upon treatment with citalopram. These findings suggest that citalopram’s anti-tumor activities on HEP-2 cells entailed stimulation of the intrinsic apoptotic pathway, which was mediated via Bcl-2 suppression.

## Background

Larynx squamous cell carcinoma is one of the most prevalent malignant head and neck tumors, with a higher incidence rate in middle-aged and elderly men worldwide [1]. In recent decades, the incidence of laryngeal squamous cell carcinoma (LSCC) has remained essentially unchanged, at around 200,000 cases per year. It has a high mortality rate of 50% and accounts for almost 1% of all cancer cases and all cancer-related deaths worldwide [2]. Despite decades of research and advances in strategies for treating patients with laryngeal cancer, overall clinical outcomes and 5-year survival rates are still unsatisfactory. The traditional therapeutic options for the treatment of LSCC mainly include partial or total laryngectomy, chemotherapy, radiotherapy, or a combination of some of these therapeutic options. Chemo-radiotherapy is considered the only treatment option for late-stage patients, although it has limited success in curing advanced cases. Successful treatment of LSCC remains a challenge due to poor patient response to radiation and/or chemotherapy and a low survival rate; therefore, an approach to new treatment strategies is required [3].

Drug repurposing is a proposed strategy for drug development for many diseases, including cancer. Due to the safety profiles of the drugs approved by the FDA and their apparent pharmacokinetics, they could be more useful compared to the discovery of new drugs [4]. Additionally, drug repurposing can be cost- and time-effective compared to traditional drug discovery methods. In the last two decades, the number of oncological patients treated with antidepressants has increased remarkably, since these patients suffer from depression and psychological stress [5]. Interestingly, since antidepressants have been used in conjunction with cytotoxic drugs, some observations have suggested that antidepressants might reduce the risk of cancer and affect the effectiveness of anticancer therapies, including colon, lung, liver, and breast cancer [6].

Selective serotonin reuptake inhibitors (SSRIs) have been shown to be cytotoxic to malignant tumors in a growing number of investigations [7]. The SSRIs zimelidine and fluoxetine have been observed to suppress the proliferation of glioblastoma, lung, prostate, and colon cancer. In addition, citalopram, clomipramine, and imipramine have also been found to induce apoptosis in myeloid leukemia and breast cancer cells [8]. Escitalopram also exhibits cytotoxic and apoptotic effects in glioma cells, according to previous reports [9]. In Burkitt’s lymphoma cells, citalopram was found to induce apoptosis accompanied by caspase activation and reverse overexpression of Bcl-2 [10]. Citalopram also demonstrated an antitumor effect against hepatocellular carcinoma cells through activation of autophagy and apoptosis via releasing cytochrome c and activating NFκB [11]. All of these results suggest that antidepressants like SSRIs may have therapeutic potential against cancer. The antitumor effect of SSRIs on human laryngeal squamous cell carcinoma is yet to be investigated. Therefore, our study was performed to explore the possible antitumor effect of citalopram as a member of the SSRIs on the human laryngeal squamous cell line (HEP-2) and to elucidate the possible molecular mechanisms underlying this action.

## Methods

### Reagents and antibodies

Citalopram, dimethyl sulfoxide (DMSO), foetal bovine serum (FBS), as well as trypan blue and MTT, were obtained from Sigma (St. Louis, USA). Dulbecco’s (DMEM) Medium as well as penicillin/streptomycin. HEPES buffer solution, gentamicin, and 0.25% trypsin–EDTA were also provided by Sigma (St. Louis, USA). The primary antibodies for B-actin, Bcl-2, BAX, cleaved caspase-9, cleaved caspase-3, Cytochrome c, and p21 were brought from Santa Cruz (Dallas, TX, USA). For Secondary antibodies, we used horseradish peroxidase-labelled immunoglobulin G (IgG) from DAKO (Darmstadt, Germany).

### Cell culture

The HEP-2 cell line was purchased from ATCC, Rockville, MD, USA. The cells were allowed to grow in DMEM with heat inactivated FBS (10%). HEPES buffer, penicillin (100 U/ml), streptomycin (100 g/ml), l-glutamine (1%), and gentamycin (50 g/ml), were added to cells, then they were kept in a humid environment at 5% CO_2_ and 37 °C.

### Cell viability assessment

Cells were seeded in a 96-well plate with a density of 1 × 10^4^ cells per well. Fresh media containing various citalopram concentrations was added 24 h after plating. The MTT test was used to calculate the viable cell yield after incubation. After replacing the medium with 100 ml of fresh medium. 10 ml of the 12 mM 3-1-2,5-diphenyltetrazolium bromide (MTT) solution was added to the well, then the plate was incubated for 4 h. After removing the medium, the formazan salt was dissolved in DMSO. Finally, the optical density was obtained at a wavelength of 550 nm. GraphPad Prism software. (San Diego, CA, USA) was used to calculate the 50% inhibitory concentration (IC50) of the drug.

### Apoptosis assay using annexin V and PI

The proportion of the apoptotic cells was further determined by annexin V and PI. HEP-2 cells were treated with citalopram, then collected after 48 h and washed twice in PBS for 20 min. HEP-2 cells were re-suspended in 100 µl of the binding buffer (10 mm HEPES/NaOH (pH 7.4), with 1 µl of FITC-annexin V added, followed by 40 min of incubation at 4 °C with propodium iodide (PI) (1 µg/ml in PBS). The cells were then examined with the BD FACS (BD Biosciences, CA).

### Cell cycle analysis

First, after seeding HEP-2 cells, the cells were synchronized before measuring the cell cycle distribution using the Cycle TESTTM PLUS DNA reagent kit. Cell synchronization achieved by serum starvation through depriving cells of growth factors that can arrest them in G1 phase. After treatment with various doses of citalopram for 48 h, the cells were harvested and collected after washing with 10 ml PBS and centrifuged 5 min at 200 × *g*. Then the supernatant removed and cells resuspended in 0.5 ml PBS and 4.5 ml pre-chilled 70% cold ethanol (−20 °C) were added in a drop wise manner to the cell suspension while vortexing to ensure fixation for all cells and avoid cell clumping. After fixation, cells were washed by PBS, then treated with RNase (DNase free). Finally, the HEP-2 cells were stained using PI dye. PI binds to DNA, and during flow cytometry, the DNA will emit a fluorescent signal which is vary depending on the amount of DNA in the cell. The cells that are in the S-phase would have more DNA than cells in G1. Cell Quest software was used to compute the cell cycle distribution.

### Western blot analysis

SDS Page was carried out as instructed. Briefly, cells were lysed in cold lysis buffer NP40 with 1:300 protease and phosphatase inhibitor cocktail tablets (Sigma/Roche, respectively) for 10 min on ice. Then, the Bradford method was used to measure the concentration of proteins in the supernatant. SDS loading buffer was mixed with 20 µg of total protein and heated to 95 °C for 10 min. After cooling, the mixture was electrophoresed by SDS PAGE using a Cleaver electrophoresis unit (BioRad). Before the incubation with primary antibodies, the membrane was blocked with 5% non-fat dried milk in TBS-T. The horseradish peroxidase-labelled secondary antibodies (Dako) were then applied to the membrane. The band intensities were evaluated after the chemiluminescent signals were recorded by a camera-based imager.

### Light microscope examination

The cells were fixed on the plate through 15 min incubation in 10% formalin at room temperature. After that, crystal violet was used to dye the cells for 20 min. After removing the stain and rinsing the panels with deionized water, they were air-dried. The morphological alterations in treated cells as compared to the control cells were captured by an inverted microscope at a 400× magnification.

### Electron microscope examination

After 48 h of treatment, the cells were harvested and the cell pellets were first fixed for 1 h in 2.5% glutaraldehyde in 0.1 M cacodylate buffer (pH 7.2) at room temperature, and post-fixed for 1 h in osmium tetroxide (1%) in the same solution. After that, the samples were washed three times (15 min each) in cacodylate buffer, dehydrated in 10% increments, and finally, ethanol series up to absolute ethanol. Using the programmable tissue processor model LEICA EM TP, fixation and dehydration processes were carried out through a graded sequence of infiltrations until the samples were lying in clean epoxy resin. The samples were then moved to a 60 °C oven for 72 h to polymerize before being allowed to reach 25 °C desiccators for 24 h. Blocks that were mounted were cut with razor blades to create a trapezoidal shape that was less than 1 mm wide and height. Using an Ultracut UCT Ultramicrotome (Leica, Austria) and a freshly prepared glass knife to assure cleanliness and sharpness, ultrathin slices (60 nm) were cut. The incredibly tiny parts were placed on 2.05 mm copper hex grids. After that, sections were doubly stained by uranyl acetate and lead citrate. A JEOL 1010 Transmission Electron Microscopy (Tokyo, Japan) was used to read stained sections at 80 kV, and a Hamamatsu digital camera C4742-57-12NR (Hamamatsu, Japan) was used to take digital pictures.

### Statistical analysis

At least three different experiments were conducted and the mean and standard deviation (SD) for each piece of data were displayed. To examine the statistical differences, the Student’s *t*-test was used. The analyses were carried out using the SPSS 21.0 software package (SPSS Inc., Chicago, IL, USA, version 21.0). The differences between the studied groups were considered to be significant at *P* ≤ 0.05.

## Results

### Citalopram decreases the cellular proliferation capacity of HEP-2 cells

**Fig. 1:**
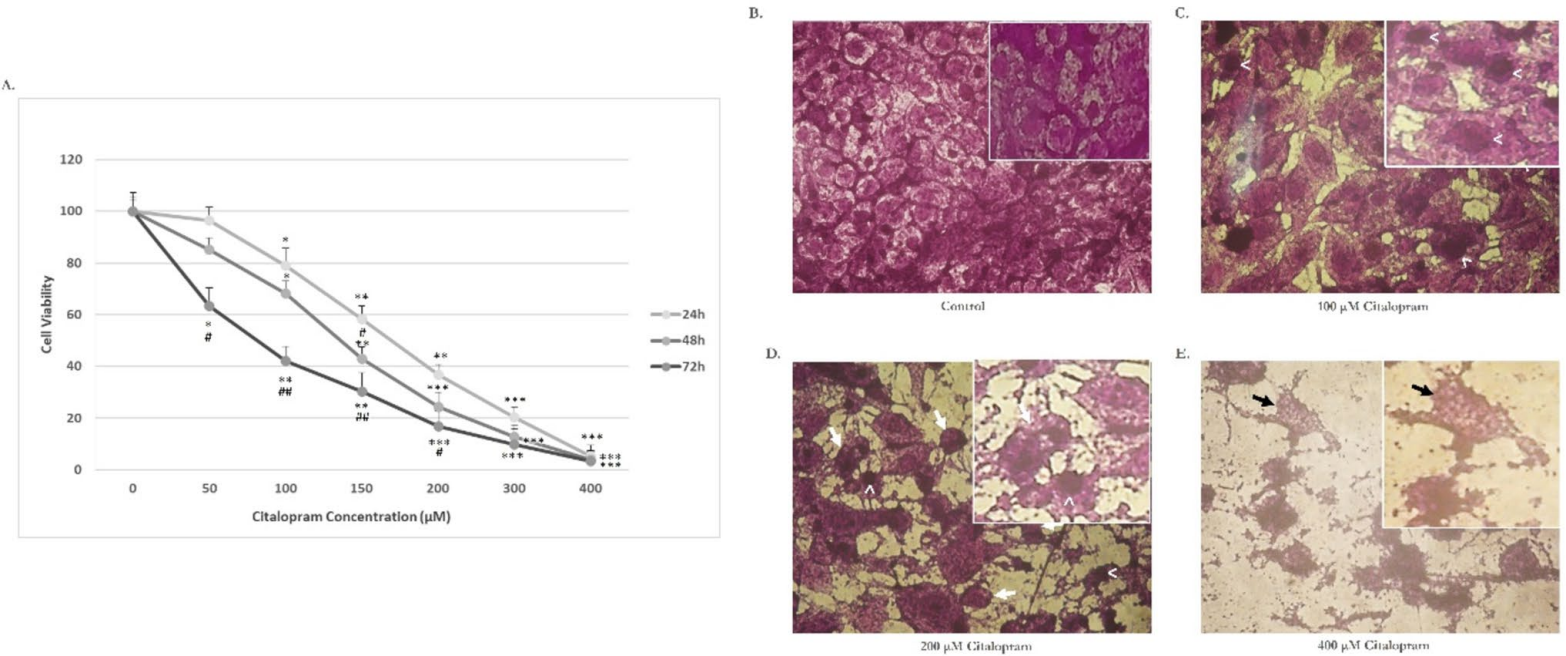
Cytotoxic effect of citalopram on Laryngeal Carcinoma HEP-2 Cell line. Cells (1 × 10^4^) were inoculated into 96-well plates and cultured in the absence or presence of citalopram (0, 50, 100, 150, 200, 300 and 400 μM) for 24 h, 48 h. and 72 h. **A** The cell viability was estimated by the MTT assay. Data are representing as mean ± standard deviation. Results are representative of three separate experiments. *P < 0.05, **P < 0.01, ***P < 0.001 versus vehicle-treated control cells, while #*P* < 0.05, ##*P* < 0.01versus corresponding dose at 24 h. **B** Morphological characteristics of untreated control cells showing normal distribution of cells with no alterations observed. Morphological changes in cells treated with, **C** 100 μM of citalopram showed a slight reduction in cell numbers with morphological alteration in the nucleus (arrowhead), **D** 200 μM of citalopram treatment showed cell shrinkage (white arrow) with obvious reduction in cell numbers and chromatin condensation (arrowhead) and **E** 400 μM of citalopram treatment showed dramatic reduction in number of cells with obvious cell lysis (black arrow) and chromatin condensation (arrowhead). Cells were observed under light microscope at ×20 magnification

The results in (Fig. 1A) show the viability of HEP-2 cells using the MTT assay. HEP-2 cells were subjected to increasing concentrations of citalopram (50, 100, 150, 200, 300, 400, and 400 μM) for 24, 48, and 72 h, whereas vehicle-treated cells were used as a control. Treatment with citalopram significantly reduced the viability of HEP-2 cells as compared to the control cells. Assessment of cell viability by MTT revealed an increase in cytotoxic effects with increasing dose and duration of citalopram treatment, reflecting a dose- and time-dependent reduction in the proliferation rate of HEP-2 cells treated with citalopram. The experimentally presented IC50 values for citalopram in HEP-2 cells were approximately 208.05 ± 7.13, 171.82 ± 4.56, and 81.6 ± 1.28 μM, at the time-points of 24 h, 48 h, and 72 h, respectively. The morphological alterations in the HEP-2 cells upon citalopram treatment were evaluated by crystal violet staining. As shown in (Fig. 1C–E) treatment of HEP-2 with citalopram resulted in a reduction of cell numbers reflecting cell deformation and the induction of cell death compared to the control non treated cells as shown in Fig. 1B). Of note, with increasing the citalopram concentration, the morphological alterations of the cells became more intensified, as shown in (Fig. 1E) of the 400 μM-treated group.

### Citalopram induces cell death in HEP-2 cells

**Fig. 2:**
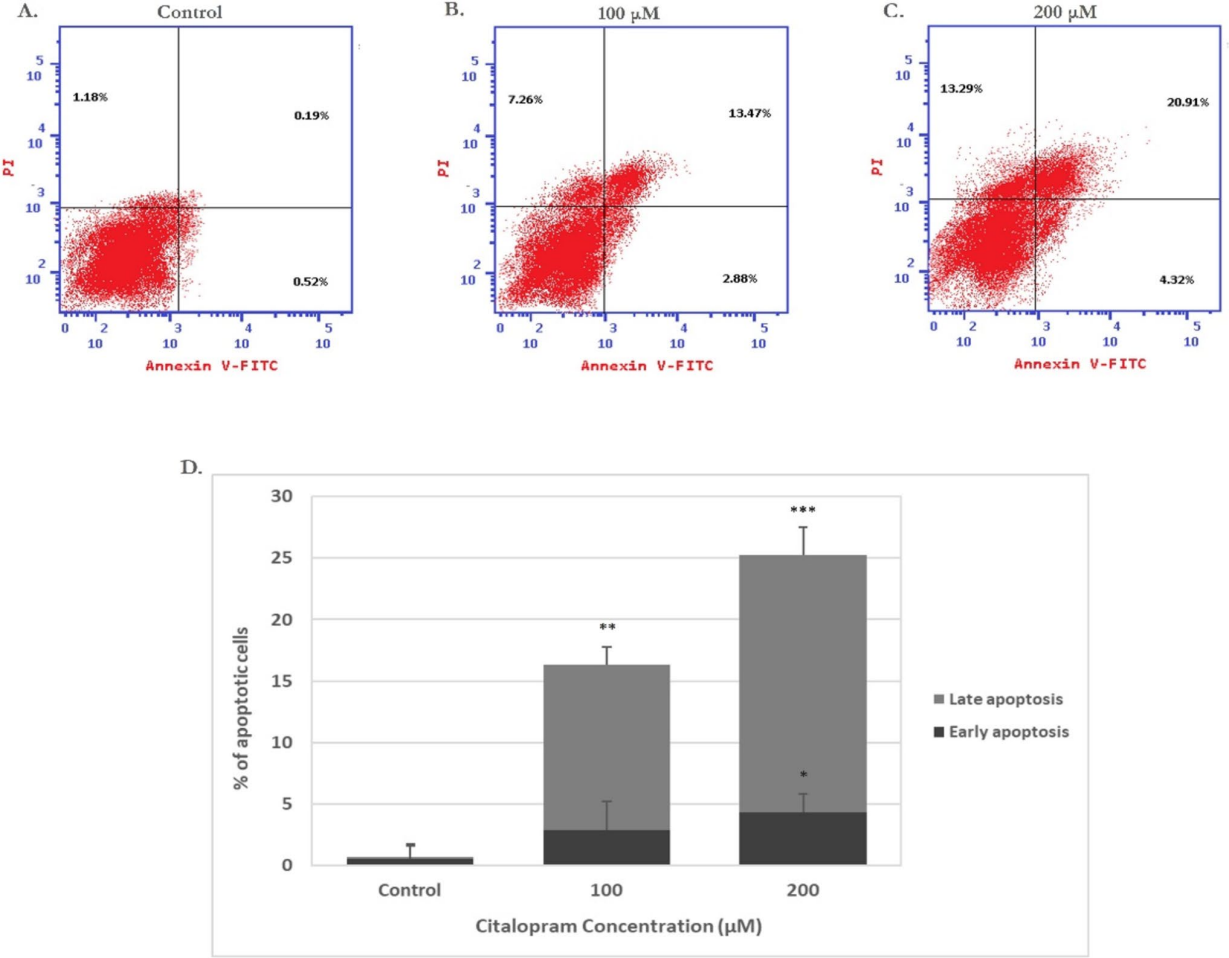
Citalopram induces apoptosis in HEP-2 cells. The annexin V FITC/PI double staining assay was performed to detect the rate of apoptosis of HEP-2 cells after citalopram treatment in **A** control untreated cells, **B** after 100 μM of citalopram treatment and **C** after 200 μM of citalopram treatment. **D** Statistical analysis of the rate of early and late apoptosis of HEP-2 cells after treatment. *P < 0.05, **P < 0.01, ***P < 0.001 versus corresponding apoptotic state in control cells. All data are expressed as mean SD from at least three independent experiments

The annexin V-propidium iodide (PI) double labelling technique was carried out in HEP-2 cells and analyzed by flow cytometry to evaluate the apoptotic effect of citalopram on HEP-2 cells. The results revealed that citalopram-induced apoptosis was dose-dependent as shown in (Fig. 2B, C) compared to control non treated cells as shown in (Fig. 2A). The percentages of cell death significantly increased from 1.89% in control cells to 23.61% and 38.52% in treated cells at 100 μM and 200 μM citalopram, respectively, as shown in (Fig. 2D).

### Citalopram induces arrest of the cell cycle at the G0/G1 phase with an increase of the sub-G1 population in HEP-2 cells

**Fig. 3:**
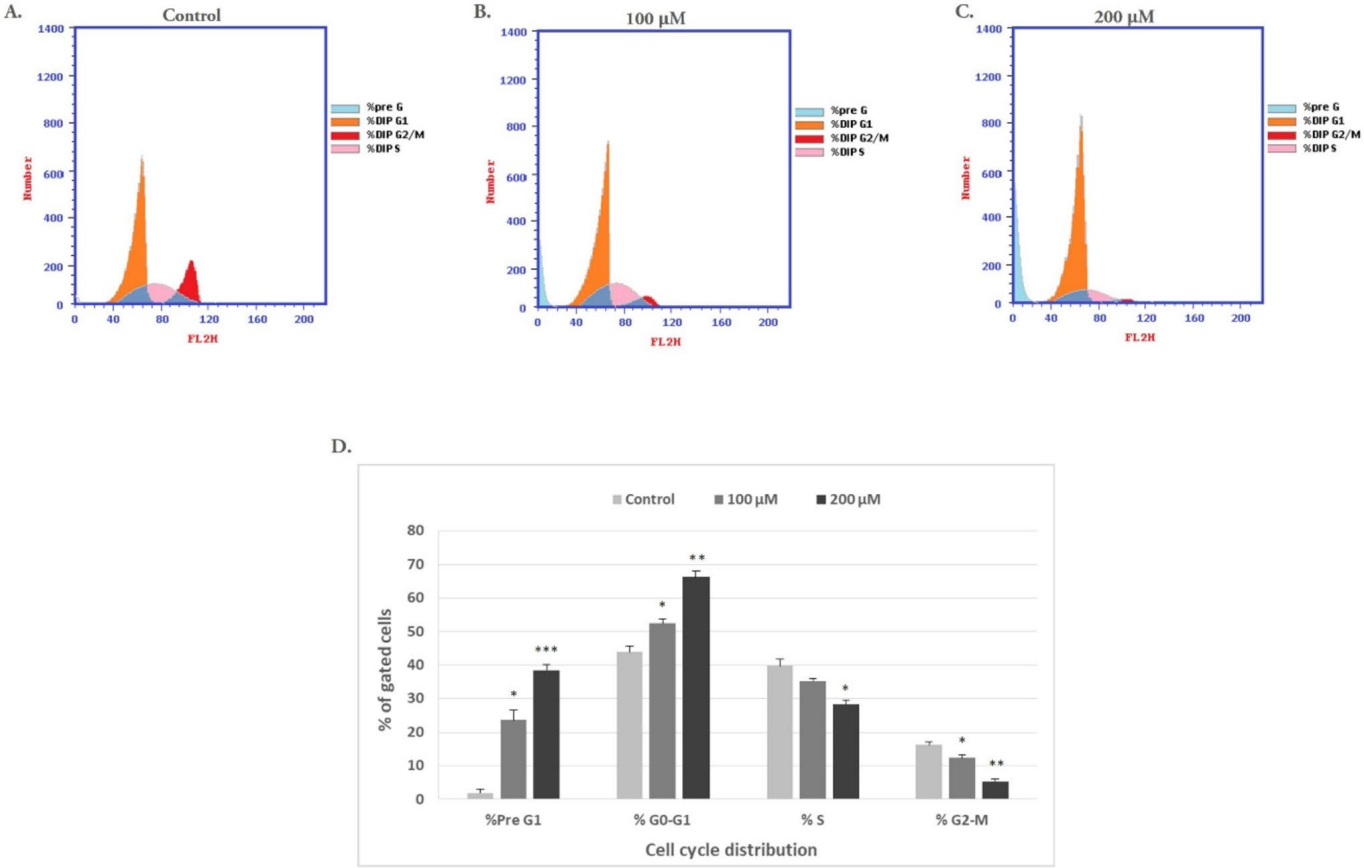
Effect of citalopram on the cell cycle distribution in HEP-2 cells. HEP-2 cells stained using PI to examine cell cycle distribution by flow cytometry with different doses of citalopram. **A **Control untreated cells, **B** 100 μM of citalopram treatment and **C** 200 μM of citalopram treatment. **D** Statistical analysis of the cell number in % of each cell cycle phase relative to the total phases. Each data point represents the SD mean of three independent experiments. *P < 0.05, **P < 0.01, ***P < 0.001 compared to control cells

**Fig. 4:**
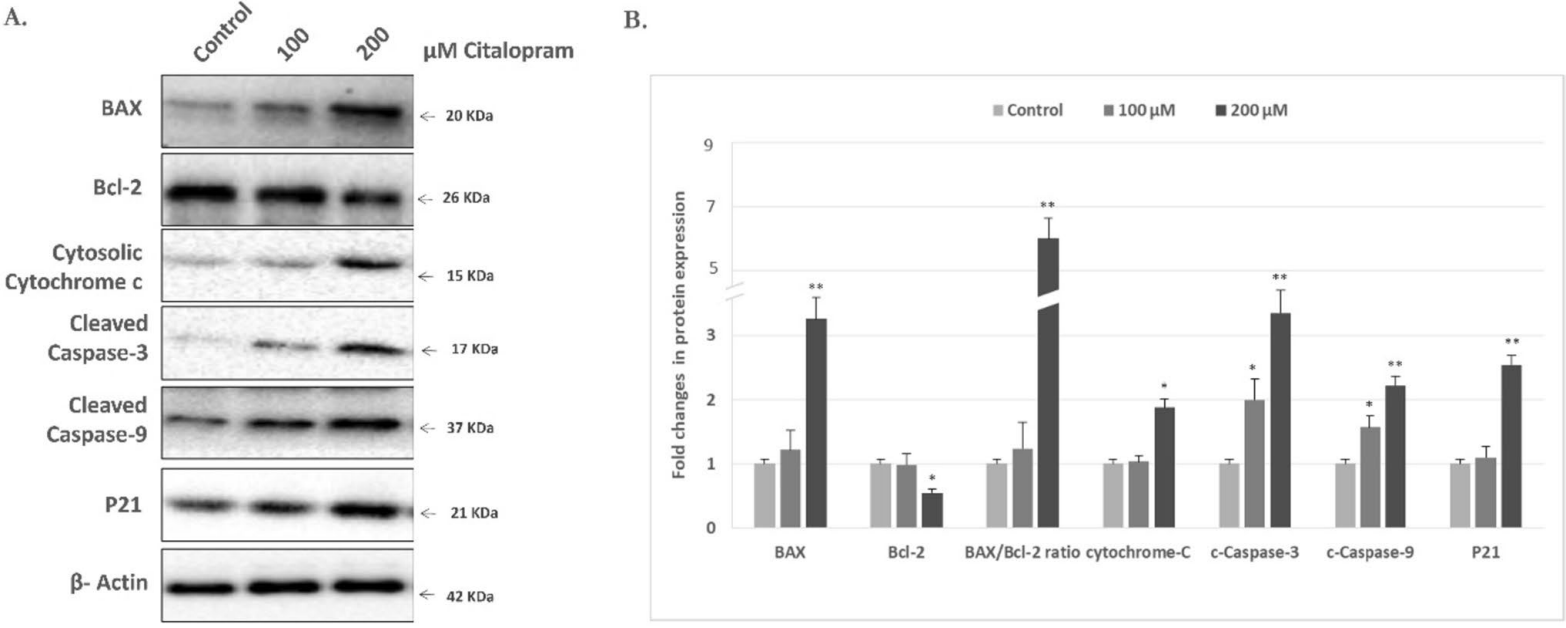
Effects of citalopram on the levels of apoptosis-related molecules. **A** Protein levels of BAX, Bcl-2, cytochrome c, cleaved caspase-9, cleaved caspase-3 and p21 in citalopram-treated HEP-2 cells were detected by western blotting. β-actine was used as a loading control. **B** The relative levels of BAX, Bcl-2, cytosolic cytochrome c, cleaved caspase-3, cleaved caspase-9 and p21were analyzed. *P < 0.05, **P < 0.01 compared with the control group. All data are presented as the mean ± SD from three independent experiments

To further evaluate the inhibitory effects of citalopram on HEP-2 cell proliferation, the cell cycle distribution was examined after treatment of HEP-2 cells with various doses of citalopram for 48 h and then stained with PI, an intercalating agent, which can be used to measure cellular DNA content in a population (Fig. 3). Incubation with 100 and 200 μM citalopram significantly increased the proportion of HEP-2 cells in the G0/G1 phase in a dose-dependent manner. In addition, the population of S-phase and G2/M-phase HEP-2 cells were significantly reduced at 100 and 200 M citalopram treatment compared to vehicle-treated control cells, as shown in (Fig. 3). In addition, changes in p21 levels can alter cell cycle progression and induce cell cycle arrest. To elucidate the mechanism by which citalopram induces cell cycle arrest, p21 levels were determined by Western blotting. The results revealed that treatment with 100 and 200 M citalopram significantly increased the p21 protein levels when compared to the control cells, as shown in Fig. 4. These results suggested that citalopram most likely potently induced cell cycle arrest by inducing the expression of p21.

### Signalling molecules involved in the citalopram-induced apoptosis in HEP-2 cells

Citalopram effectively induces the apoptotic death of HEP-2 cells. Thus, the apoptotic pathway involved was further dissected. The levels of cleaved caspase-3 and caspase-9 protein expression showed a dose-dependent, significant increase in citalopram-treated HEP-2 cells, as shown in (Fig. 4). In addition, the upstream molecule of caspase-9, cytochrome c level, was further studied. The results revealed that the level of cytoplasmic cytochrome c expression was significantly higher in citalopram-treated cells than in the control group (Fig. 4). These data revealed that citalopram-induced apoptosis of HEP-2 cells occurs via a signalling pathway that is mediated by mitochondria.

### Effects of citalopram on the upstream regulators of the mitochondrial apoptotic signalling pathway

To confirm that citalopram induces apoptosis and triggers the mitochondrial apoptotic pathway in HEP-2 cells, protein levels of the apoptotic regulators BAX (pro-apoptotic) and Bcl-2 (anti-apoptotic) were determined by Western blotting. The BAX/Bcl-2 ratio was also calculated, which is considered the molecular switch that initiates apoptosis. The current results indicated decreased levels of Bcl-2 accompanied by elevated BAX levels in citalopram-treated HEP-2 cells when compared to the control cells. The BAX/Bcl-2 ratios in citalopram-treated HEP-2 cells were significantly higher than the control cells, as shown in (Fig. 4).

### Ultra-structural changes in HEP-2 cells

**Fig. 5:**
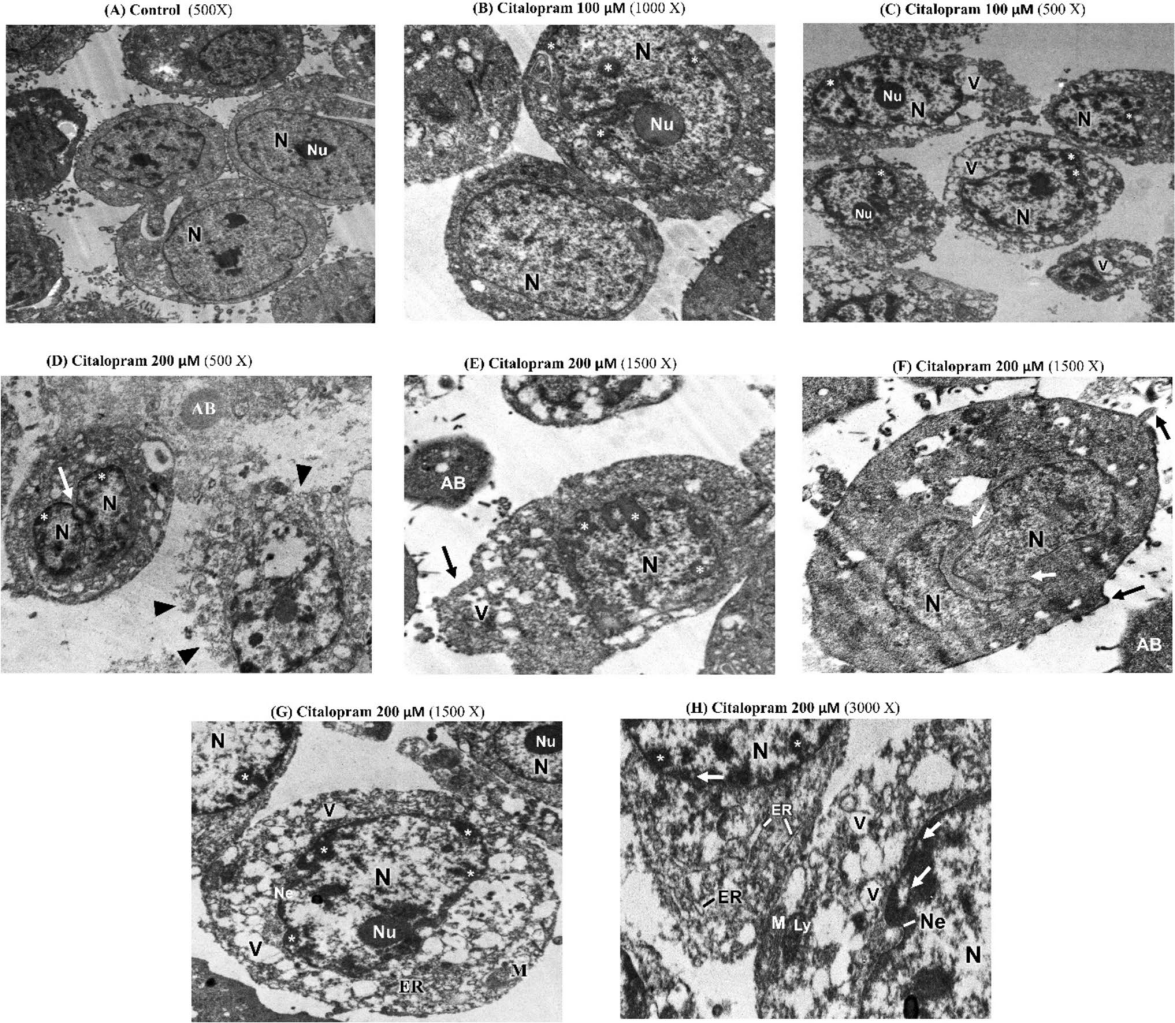
Ultrastructural alterations in HEp-2 cells treated with citalopram for 48 h under transmission electron microscopy. **A** Control HEP-2 cells showing normal morphology. **B** HEp-2 cells treated with citalopram showing cytoplasmic and nuclear granulation and chromatin condensation (asterisks). **C** Vacuole formation (V), peripheral chromatin condensation (asterisks) and appearance of moderate apoptotic bodies were observed. **D** Treated HEP-2 cells releasing its fragmented cytoplasmic contents into the sinusoidal spaces (head arrow) and apoptotic bodies (AB). **E** Separate aggregates of chromatin observed (asterisks), vacuoles formation (V) membrane blebbing (black arrow) and apoptotic bodies (AB). **F** Treated cells showed nuclear fragmentation (white arrow) and membrane blebbing (black arrow). **G** Irregular nucleus (N) surrounded by a slightly invaginated nuclear envelope (Ne), marginated nucleolus (Nu), remarked vacuolation, swelled ER and deformative mitochondria (M) observed. **H** Treated cells showed moderate nuclear envelope dilatation (Ne), ring chromatin condensation “spheridium” (white arrow), lysosome (Ly) and swelled ER (Nu), remarked vacuolation, swelled ER and deformative mitochondria (M)

A transmission electron microscope (TEM) was applied to monitor the ultrastructural changes in HEP2 cells after citalopram treatment with different doses (100 and 200 M). After 48 h of treatment, as shown in Fig. 5A, control HEP-2 cells showed normal morphology. The control cells had an irregular oval shape with a well-defined cell membrane, large round or ellipsoidal nuclei with an electron-dense nucleolus, well-preserved euchromatin, and a visible nuclear envelope with some invaginations (Fig. 5A). The cells treated with citalopram showed that death corresponded very well to the classic signs of apoptosis: increased cell granularity, cell shrinkage, and the formation of apoptotic bodies (Fig. 5B, C). Interestingly, citalopram showed maximal apoptosis, with a higher citalopram concentration (200 µM) being observed (Fig. 5D–H). This evidence suggests that the apoptogenic effects of citalopram on HEP2 cells are dose-dependent. The initial induction of cell death by citalopram involved cell shrinkage (Fig. 5C) and chromatin condensation, cracking, and migration towards the nuclear periphery, and the cytoplasm began to granulate (Fig. 5C–G). Some cells showed chromatin clumps marginalized in congested nuclear regions (Fig. 5E). The nucleus generates numerous compact electron-dense micronuclei (Fig. 5B, C), nuclear envelope dilation (Fig. 5H), and cell membrane blebbing (Fig. 5E, F), which were the next stages in this sequence of apoptosis, followed by nuclear fragmentation (Fig. 5D, F), and the continuous vesicle phase. Other changes, including deformed mitochondria and a reduced number of cytoplasmic organelles, were also observed (Fig. 5B–H). Some cells had slight or extensive vacuolation in the cytoplasm, with the vacuoles being bound by a single membrane layer (Fig. 5C, G, H). It was observed that HEP2 cells treated with a higher concentration of citalopram showed late-stage apoptotic phenomena that included the formation of apoptotic bodies (Fig. 5D, E). HEP2 cells treated with citalopram showed similar morphological aspects of apoptosis, but necrotic features were also observed. Necrotic cells showed rapid permeabilization of the plasma membrane. It shows progressive discontinuities causing general cellular hydration, swelling, and organelle destruction (Fig. 5D). Cytosolic components are released into the extracellular space through a damaged plasma membrane (Fig. 5D).

## Discussion

In the current study, we examined the cytotoxic effects of citalopram against HEP-2 cells and elucidated the mechanism underlying its effects. Several studies have suggested that the use of neuroleptic medications as a drug repurposing strategy may lessen the chance of developing specific types of cancer [7]. On the other hand, little is understood regarding the mechanism by which SSRI causes LSCC cell death. In this work, we elucidated the mechanisms through which citalopram induces cell death in human laryngeal squamous carcinoma HEP-2 cells.

It is well recognized that unchecked proliferation plays a crucial role in the growth of tumors [12]. Therefore, compounds that induce cell cycle arrest, cellular death, and tumor cell inhibition are valuable in cancer research. Apoptosis has long been identified as a deterrent to carcinogenesis. Its stimulation is one of the primary mechanisms preventing the spread of cancer and is crucial for the testing of novel anticancer medications [13]. In this study, citalopram was reported to significantly increase the proportion of apoptotic cells in the HEP-2 cell line.

The intricate mechanics of apoptosis involve mitochondria, which start the intrinsic pathway of apoptosis [14]. During the initial stages of apoptosis, it has been shown that a certain group of proteins that are typically found in the mitochondrial intermembrane space are released. Among these important proteins are Smac/DIABLO, adenylate kinase-2 (AK-2), and cytochrome c [15, 16]. Cytochrome c is located in the mitochondrial intermembrane space. The mitochondrial release of cytochrome c is a crucial stage in the apoptotic process [17, 18]. Cytochrome c interacts with the adaptor molecule, apoptotic protease activating factor 1 (Apaf-1), leading to the maturation of pro-caspase-9 into caspase-9, which subsequently cleaves and activates pro-caspases-3 and -7 [19, 20]. These effector caspases are in charge of cleaving many proteins, which produce the biochemical as well as morphological characteristics of apoptosis [21]. Consistent with this scenario, our results indicated a significant increase in the protein expression of cytochrome c concomitant with increased expression of cleaved caspase-3 and caspase-9 was significantly upregulated in cells upon citalopram treatment, suggesting involvement of the intrinsic apoptotic pathway.

The Bcl-2 protein family’s members are key components of apoptosis. According to reports, Bcl-2 and the pro-apoptotic component BAX can create a heterodimer that can inhibit BAX’s pro-apoptotic activity. Consequently, Bcl-2 overexpression causes apoptotic resistance. The ratio of BAX/Bcl-2 has a crucial role in determining the fate of cells, whether they undergo apoptosis or not [22]. In the current study, a decrease in Bcl-2 protein expression was found in citalopram-treated HEP-2 cells. In contrast, the protein level of BAX in HEP-2 cells was upregulated after citalopram treatment, thereby increasing BAX/Bcl-2 ratios in HEP-2 cells.

According to Elmore, apoptosis is an organized, energy-required process that is essential for tissue survival and homeostasis [23]. The morphological signs of apoptosis include cell shrinkage, membrane bleb formation, nuclear condensation, and the development of pyknotic or apoptotic bodies [24, 25]. Citalopram has been shown to cause morphological changes in HEP-2 cells, including the development of apoptotic bodies and progressive chromatin condensation. During the late stages of apoptosis, the nucleus continues to condense and disintegrate within a cell with an intact membrane [24, 25]. These morphological signs can be initiated by the cleavage of various proteins by caspase activation [26], which is upregulated upon treatment with citalopram in HEP-2 cells. Nuclear proteins of the mitotic apparatus (NuMA) and lamin proteins preserve the structural integrity of the nucleus [27, 28]. Both can be cleaved by caspase-3 [29], which are highly expressed when HEP-2 cells are treated with citalopram.

Loss of proper mammalian cell cycle control drives cellular transformation. Inhibitors of cyclin-dependent kinase (CDK) are essential for regulating cell cycle progression. By encouraging cell cycle arrest in response to diverse stimuli, p21 is known to inhibit tumor growth. Evidence from biochemical and molecular studies suggests that p21 acts as a major effector of various tumour suppressor pathways to stimulate anti-proliferative activities [13]. The p21 protein expression in this study was determined after treatment with citalopram, and it was observed to be significantly upregulated in cells treated with citalopram. The significant increase in p21 observed after citalopram treatment may clarify the mechanism by which citalopram induces a cell cycle arrest in HEP-2 cells at the G0/G1 phase. This is consistent with a previous study reporting that p21 induces G0/G1 as well as G2/M arrest through the inhibition of CDK2 activity and the blocking of CDK1 activity, respectively [30].

## Summary of the work

See Fig. 6.

**Fig. 6:**
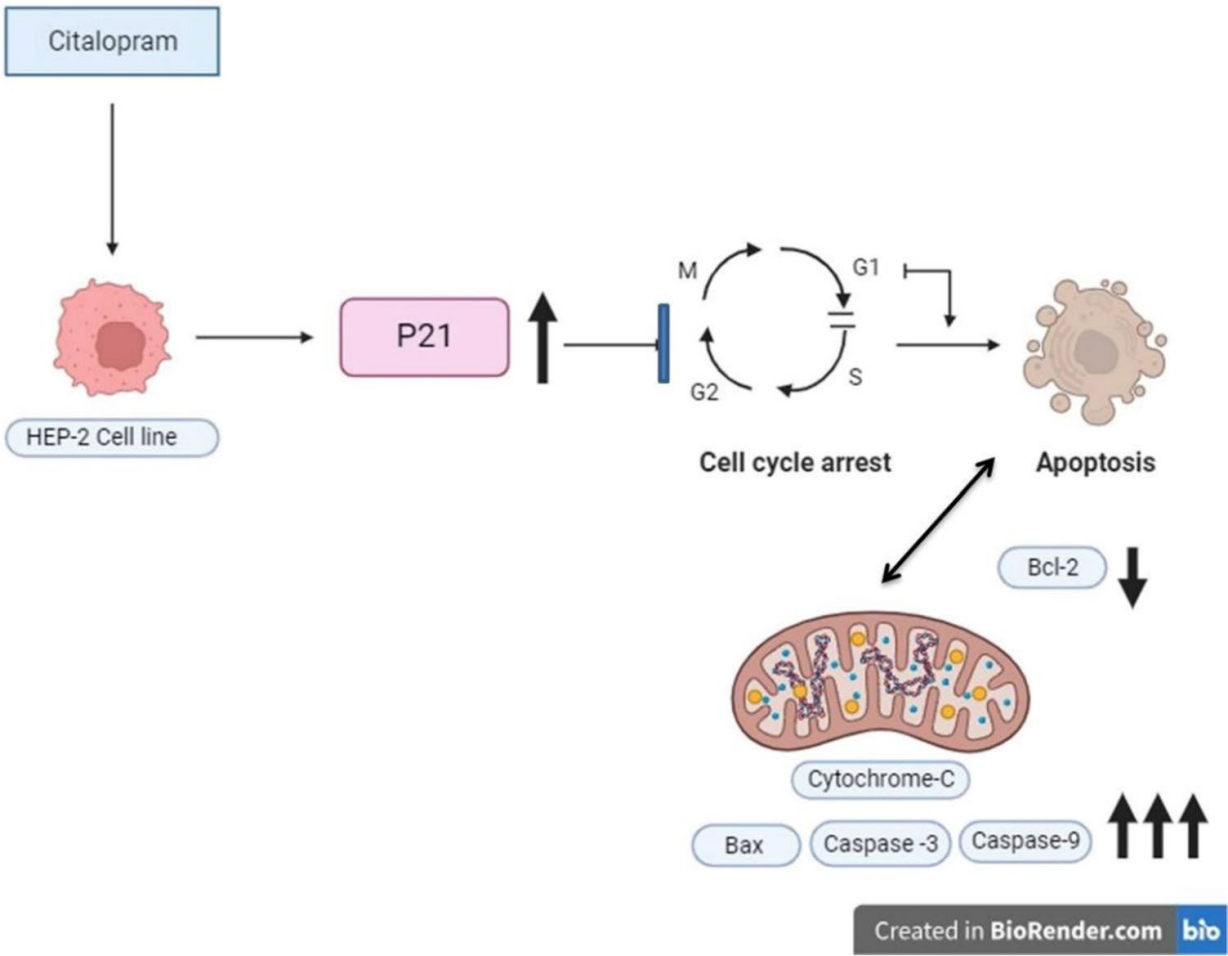
Citalopram as a promising candidate with anticancer properties against HEP-2 cells. Citalopram induces G1/G0 phase cell cycle arrest by overexpression of P21. Moreover, it activates intrinsic apoptotic pathway by down regulation of Bcl-2 and activation of BAX resulting in release of cytochrome c to cytoplasm and activation of caspase3 and 9. (Schematic drawing created with BioRender.com)

## Conclusions

Here, we suggest citalopram as a promising candidate with anticancer properties against HEP-2 cells via activation of intrinsic apoptotic pathway and inducing cell cycle arrest at the G0/G1 phase.

## Limitations

This study elucidated the antitumor potential of citalopram against HEP-2 cells. However, the combinatorial cytotoxic effect of citalopram with traditional chemotherapeutic agents is required to develop citalopram as a promising candidate for the treatment of LSCC. Moreover, in vivo studies are required to evaluate the cytotoxic effect of citalopram on normal tissue in different organs.

## Data Availability

No datasets were generated or analysed during the current study.

## Abbreviations

LSCC: Human laryngeal squamous carcinoma
SSRIs: Selective serotonin reuptake inhibitors
BAX: Bcl-2-associated X factor
BCL-2: B-cell lymphoma 2 protein
FBS: Foetal bovine serum
PBS: Phosphate buffered saline
DMSO: Dimethyl sulfoxide
PI: Propodium iodide
MTT: 3-(4,5-Dimethylthiazol-2-yl)-2,5-diphenyltetrazolium bromide
IC50: Half-maximal inhibitory concentration
SDS PAGE: Sodium dodecyl sulfate polyacrylamide gel electrophoresis
CDK: Cyclin-dependent kinase
